# Quantitative Analysis of Protein Fouling in Virus Removal Filtration Membranes Through Electron Tomography

**DOI:** 10.3390/membranes15120369

**Published:** 2025-12-02

**Authors:** Mohammad A. Afzal, Kaitlyn P. Brickey, Enrique D. Gomez, Andrew L. Zydney

**Affiliations:** 1 Department of Chemical Engineering, The Pennsylvania State University, University Park, PA 16802, USA; maa6831@psu.edu (M.A.A.); kbp69@psu.edu (K.P.B.); edg12@psu.edu (E.D.G.); 2 Department of Materials Science and Engineering, The Pennsylvania State University, University Park, PA 16802, USA; 3 Materials Research Institute, The Pennsylvania State University, University Park, PA 16802, USA

**Keywords:** virus filtration, protein fouling, pore morphology, electron tomography, pore size distribution

## Abstract

Protein fouling can significantly reduce the filtrate flux, capacity, and virus retention during processing of plasma- or mammalian cell-derived biopharmaceuticals through virus removal filters. We use focused ion beam (FIB) milling and scanning electron microscopy (SEM) to directly evaluate changes in 3D pore structure in a Viresolve^®^ Pro membrane due to fouling by human serum immunoglobulin G. Protein fouling causes a significant reduction in the membrane porosity, which decreases by approximately 40% in the size-selective region near the exit of the highly asymmetric Viresolve^®^ Pro membrane after the filter is fouled to 90% flux decline. There is a corresponding reduction in the number of small pores by more than a factor of two. Model simulations of flow and particle transport in the protein-fouled membrane are in good agreement with independent experimental measurements of the permeability and location of particle capture. Simulations show an upstream shift in the location of nanoparticle capture (away from the filter exit) by about 0.4 µm for the membrane fouled to 90% flux decline. This is due to pore constriction from protein deposition, highlighting how fouling redistributes flow paths within the membrane. These results demonstrate the capability of using FIB-SEM to directly evaluate the effects of protein fouling on the 3D pore structure in virus removal filters, providing important insights into how protein fouling alters the performance of these highly selective membranes.

## 1. Introduction

Protein fouling represents one of the most significant challenges in virus filtration as it affects both the filtrate flux and capacity of the virus filter as well as the degree of virus removal [1,2,3,4,5,6]. Several studies have investigated the mechanisms governing protein fouling and flux decline in commercial virus removal filters. Bieberbach et al. [7] reported that some monoclonal antibodies cause irreversible fouling of the Virosart HC virus filter, likely due to the presence of small aggregates, while others cause a reversible flux decline attributable to reversible oligomer formation. Suh et al. [8] compared the fouling behavior of seven commercial virus removal filters using bovine serum albumin as a model foulant. The most rapid flux decline was obtained with the highly asymmetric Viresolve^®^ NFP membrane, with a capacity of less than 100 L/m^2^, while the Planova^TM^ BioEX and 20N membranes show less than a 15% flux decline after a volumetric throughput of more than 250 L/m^2^. Analysis of the flux decline data revealed that the behavior of the Viresolve^®^ NFP and Pegasus™ SV4 membranes was best described by the pore-constriction model, while the Viresolve^®^ Pro and Virosart^®^ CPV membranes follow a complete pore-blockage mechanism. These studies demonstrate that the dominant fouling mechanism is closely linked to both membrane morphology and protein characteristics. Peles et al. [9,10] further evaluated the effect of transmembrane pressure on IgG fouling in the Viresolve^®^ Pro membrane, showing that the flux decline was well described by a sequential pore-blockage and cake-filtration model with fouling parameters strongly dependent on pressure.

Several research groups have shown that protein fouling can significantly compromise virus removal. Bolton et al. [2] reported that the log reduction value (LRV) for the Viresolve^®^ NFP filter declines by 4-logs after less than 70 L/m^2^ filtration of 1 g/L IgG solutions, with the data well described using a simple model in which protein fouling occurs preferentially in the smallest pores, thereby redirecting the flow to the larger, less retentive, pores. In contrast, Stuckey et al. [11] evaluated virus retention for the Planova^TM^ 20N virus removal filter and found no correlation with the extent of fouling. Afzal et al. [1] found a significant decay in virus retention for the Ultipor^®^ DV20, Pegasus^TM^ SV4, and Viresolve^®^ NFP membranes during filtration of 1 g/L IgG solutions, with a smaller decay in virus retention for the Viresolve^®^ Pro membrane. The data for the two relatively homogeneous virus filters (the Ultipor^®^ DV20 and Pegasus^TM^ SV4) appear to collapse to a single curve when plotted as a function of the flux decay, but very different behavior was seen with the highly asymmetric Viresolve^®^ NFP and Viresolve^®^ Pro membranes. This highlights that the impact of fouling on virus retention depends strongly on the membrane morphology and the location of particle capture within the membrane.

Although there have been no direct studies of the changes in pore size or porosity due to protein fouling of virus removal filters, some insights into these phenomena have been obtained by evaluating the effects of protein fouling on the location of virus or nanoparticle capture. Leisi et al. [4] used confocal microscopy to examine the capture location of fluorescently labeled Minute Virus of Mice (MVM) in the presence and absence of IgG. The labeled virus was captured closer to the entrance of the Planova^TM^ 20N membrane when the virus filtration was performed in the presence of IgG, which was likely due to constriction of the pores due to protein fouling. Interestingly, the opposite behavior was seen with the Pegasus^TM^ SV4, with the virus penetrating deeper into the filter. Fallahianbijan et al. [12] examined the capture of gold nanoparticles within the highly asymmetric Viresolve^®^ Pro and Viresolve^®^ NFP membranes using scanning electron microscopy. Fouling by IgG caused the gold nanoparticles to be captured further upstream (closer to the entrance) in the Viresolve^®^ Pro membrane, but protein fouling had no apparent effect on nanoparticle capture in the Viresolve^®^ NFP membrane. The basis for these differences in behavior are unclear, in part because there have been no direct 3D visualizations of the pore structure or flow characteristics of fouled nanoporous membranes.

Here, we directly investigate for the first time how protein fouling affects the internal pore structure of the Viresolve^®^ Pro membrane using focused ion beam scanning electron microscopy (FIB-SEM) to obtain nanometer-resolution 3D reconstructions of the pore space. FIB-SEM is one of the few methods that has been shown to provide accurate characterization of the 3D pore space in clean (pristine) membranes [13,14,15,16,17,18]. The Viresolve^®^ Pro membrane is one of the most widely used virus removal filters in biopharmaceutical manufacturing. In addition, there has been significant prior work on both the virus retention characteristics and 3D pore space for the clean membrane [1,9,10] that have demonstrated virus retention in a relatively thin region near the exit of the highly asymmetric structure that can be readily studied by FIB-SEM. To quantify the effects of protein fouling, the reconstructed pore volumes for the clean and fouled membranes were used to numerically simulate fluid flow and virus transport/retention in these membranes. This provided insights into how changes in the pore space influence membrane permeability and virus capture behavior. Altogether, our work provides new understanding of how fouling alters the membrane pore structure and how these changes ultimately impact filtration performance.

## 2. Materials and Methods

### 2.1. Membrane Fouling

Experiments were performed with a single-layer asymmetric Viresolve^®^ Pro membrane using human serum immunoglobulin G (IgG) as a model protein. IgG was obtained from Nova Biologics (Oceanside, CA, USA) as a lyophilized powder, dissolved in phosphate-buffered saline (PBS) to the desired concentration, and then filtered through a 0.2 µm first and then a 0.1 µm pore size membrane to remove any aggregates and undissolved protein.

The polyethersulfone Viresolve^®^ Pro membranes were provided by MilliporeSigma (Bedford, MA, USA). The membranes were cut into 25 mm diameter disks and placed in a polypropylene filter holder (43303010; Advantec MFS, Inc., Dublin, CA, USA) providing an effective filtration area of 3.5 cm^2^. The total membrane thickness is approximately 140 µm based on SEM cross-sections previously reported for the same membrane [12]. The shiny side (size-selective skin) of the membrane was placed facing away from the feed. The membranes were flushed with 70 L/m^2^ of PBS, and the membrane permeability was evaluated from the buffer flux measured at different pressures. The permeability (reported in units of L m^−2^ h^−1^ kPa^−1^) corresponds to the slope of the linear flux–pressure relationship.

All fouling experiments were performed under constant pressure operation at 210 kPa. Membranes were fouled by challenging with 0.1 or 1 g/L solutions of IgG in PBS until the filtrate flux had declined by a pre-specified target (either 40% or 90%). After fouling, membrane samples were air-dried for 24 h in a Petri dish before analysis by FIB-SEM. SEM images for gold nanoparticle capture in the clean and fouled Viresolve^®^ Pro membranes were obtained by Fallahianbijan et al. [12].

### 2.2. FIB-SEM Tomography

Sections from both fouled and unfouled membranes were cut into 5 × 5 mm squares and placed on SEM stubs with the shiny side facing upward. Prior to imaging, all samples were sputter-coated with a 5 nm iridium layer using the standard iridium protocol for the Leica EM ACE600 sputter coater (Leica Microsystems, Wetzlar, Germany); this minimized charging effects during imaging. Images were obtained with a dual-beam FEI Helios 660 FIB-SEM (Thermo Fisher Scientific, Waltham, MA, USA) in the Materials Characterization Laboratory at Penn State. During tomography, the sample stage was tilted to 52°, with the membrane surface aligned perpendicular to the milling direction.

First, a 100 nm-thick carbon layer was deposited using the electron beam, followed by a 1 µm-thick carbon layer using the ion beam to protect the membrane from subsequent damage. The location for subsequent milling/observation was chosen randomly, with milling performed using an ion beam at 30 kV and 24 pA to expose a clean cross-section of the membrane. Fiducial markers were introduced to facilitate alignment of both the ion and electron beams. SEM images were obtained sequentially, with 7 nm thick slices of the membrane material removed by ion beam milling between each image. This resulted in approximately 150–200 slices per dataset.

The FIB-SEM slices often showed lateral and vertical drift after image acquisition due to small shifts in the stage, sample charging, or beam intensity over time. To correct for this drift, the uniform carbon protective layer, which appears at the top of each slice, was used as a stable reference across the entire stack of images. Initial alignment attempts using standard methods in Avizo software, Version 2024.2 (Thermo Fisher Scientific, Waltham, MA, USA), such as least-squares and gravity-based alignment, introduced noticeable distortion of pore structure, as discussed in the Supplementary Information. To address this, vertical alignment was performed using a custom-written plugin [19] in ImageJ software, Version 1.54j [20], with the carbon deposition layer serving as a consistent template across all slices. Once vertical alignment was complete, horizontal drift was manually corrected in Avizo by overlaying adjacent slices and ensuring continuity of pore features across the sample depth. This two-step approach maintained the natural pore orientation, avoided artificial cropping, and protected the full depth of the fouling zone [21]. The effectiveness of the alignment procedure was qualitatively assessed by comparing the final reconstruction with results obtained using Avizo’s standard alignment methods (Figure S1), which exhibited compression artifacts that were not present in the ImageJ-based reconstruction.

Following alignment, the image stack was processed in Avizo to enhance contrast prior to segmenting the porous regions from the surrounding solid material. Because the image brightness was highly variable due to protein fouling, an adaptive histogram equalization was applied to improve contrast in darker regions. All reconstructions were then imported into GeoDict^®^ (Math2Market GmbH, Kaiserslautern, Germany), where a segmentation workflow was applied for both the clean and fouled membranes. The structure was split into two zones (upper and lower) and segmented separately. Local contrast enhancement and independent thresholding were applied to each subregion. After segmentation, the two subregions were merged into a single reconstruction using the ‘Attach’ function in GeoDict^®^.

Porosity and pore size distributions were calculated using a custom MATLAB (Version 24.2) script. Porosity was calculated as the number of pore voxels divided by the total number of voxels in any given region of the image. The pore size distribution was evaluated from binary 3D reconstructions (pores = 1, solid = 0) using the ‘bwdist’ function to generate a Euclidean distance map. This map stores, for each pore voxel, the shortest distance to the nearest solid boundary, which represents the radius of the largest sphere that can fit at each point inside the pore space. The pore diameter was calculated by doubling the distance value and multiplying by the voxel size (7 nm).

### 2.3. Fluid Flow and Particle Transport

Flow and particle capture simulations were carried out on the segmented 3D volumes using the FlowDict and FilterDict modules in GeoDict^®^. The flow field was evaluated by solving the steady-state incompressible Stokes flow equations within the segmented domain, with the velocity field updated after each batch of challenge particles. No-slip conditions were imposed at all pore walls, and symmetry boundary conditions were used on the lateral faces. The computational domain corresponded directly to the segmented FIB-SEM volume, with open inlet and outlet boundaries. Since GeoDict^®^ evaluates the velocity field for a fixed inlet pressure and velocity, an iteration was performed on the inlet velocity to match the transmembrane pressure drop of 210 kPa (30 psi) used in the experiments (within a relative error of less than 10^−3^ corresponding to 0.21 kPa).

Particle transport was evaluated using the FilterDict module, which incorporates both hydrodynamic drag and Brownian motion; electrostatic and hydrophobic interactions were not included in the analysis. The filter was challenged with 20 nm spherical nanoparticles at a concentration of 10^8^ particles/mL. Particle-solid interactions were governed by GeoDict’s built-in Hamaker collision model, with the Hamaker constant set to a negligibly small value (10^−200^). Particle capture occurred when the particle velocity became negligible (<<1 nm/s) when contacting the pore surface, corresponding to geometric confinement.

The particle challenge was performed in discrete batches of 25 particles. This involved (i) evaluating the steady Stokes flow field through the current pore geometry, (ii) injecting and tracking the motion/capture of 25 particles through that pore geometry, and (iii) updating the pore structure based on the location of captured particles. After each batch, the locations where particles had been captured were mapped onto the voxelized geometry. Voxels in which the particle volume fraction exceeded the default blocking threshold (0.5) were converted from pore space to solid, with the flow field recomputed on this updated structure before challenging with the next particle batch. Thirty batches (750 total particles) were simulated in total, taking 6–10 h of computational time, which provided stable capture statistics while keeping the overall computation time manageable.

## 3. Results and Discussions

### 3.1. FIB-SEM Tomography

**Figure 1:**
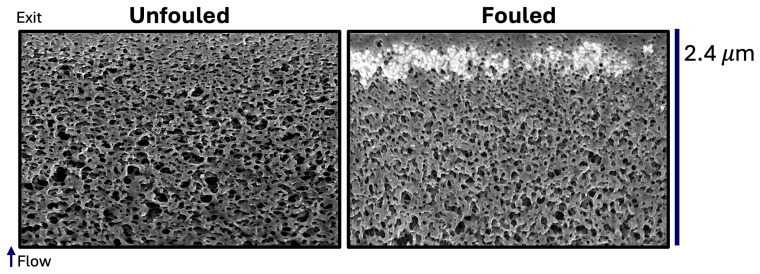
Representative SEM cross-sectional slices of the Viresolve^®^ Pro membrane comparing an unfouled control (**left**) and a fouled membrane (**right**) after filtration with IgG until a 40% flux decay. The flow direction is from bottom (inlet of reconstruction) to top (exit). Both images were acquired at 5 kV and 200 pA.

Figure 1 shows representative images of 2D slices through the Viresolve^®^ Pro membrane, with the clean (unfouled) membrane shown on the left and a membrane fouled with a 1 g/L solution of IgG at a constant pressure of 210 kPa until a 40% flux decay shown on the right. In each image, the flow is from bottom to top, with the top surface corresponding to the size-selective skin at the filter exit. The clean Viresolve^®^ Pro membrane shows an asymmetric structure, with larger, more open pores near the bottom and a tighter, more constricted region near the exit. Protein fouling is readily visible in the right image by the bright white regions near the upper surface, i.e., just below the exit of the membrane. This is due to the deposition of IgG (and IgG oligomers) in the small pores in this region of the membrane.

**Figure 2:**
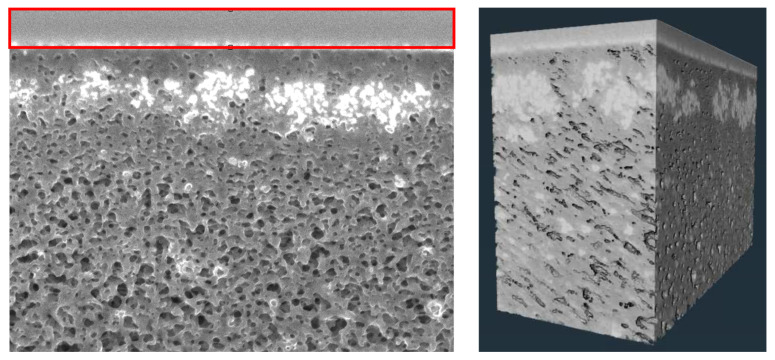
Cross-sectional (**left**) and 3D (**right**) images of the protein-fouled Viresolve^®^ Pro membrane. The red box in the left panel shows the flat carbon layer deposited on the exit surface of the membrane (before milling) that served as a template for vertical alignment.

Initial images were obtained at 5 kV and 200 pA, but a significant loss of IgG was observed during repeated scans at the same location (without ion beam milling); this is shown in Figure S3 in the Supplementary Information. Several efforts were made to optimize the scanning conditions, with a significant improvement in image quality without loss of IgG obtained by reducing the current to 25 pA and using a 7 nm slice thickness. Under these conditions, no visible loss of protein contrast or structural distortion was observed in sequential scans of the same region, and the morphology and brightness of the protein-rich zones remained consistent throughout image acquisition, thus preserving the fouling layer during tomography. Vertical alignment of the slices was performed in ImageJ with the carbon deposition layer at the top of the image serving as a consistent template across all slices, as shown in Figure 2. The resulting 3D reconstruction (right panel) retains geometric accuracy without the compression issues that were introduced when using a least-squares alignment.

**Figure 3:**
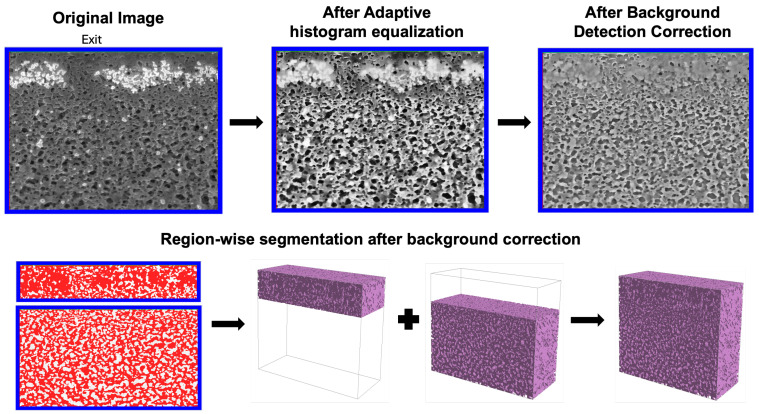
Image processing and segmentation workflow for a fouled membrane. The top row shows the original grayscale image, followed by adaptive histogram equalization and background correction. The bottom row shows the segmentation of the upper and lower regions and the assembly of the final 3D reconstruction.

Figure 3 shows the workflow used to develop the 3D reconstructions of the pore space within the virus removal filter. The contrast in the original image (left) was enhanced by applying an adaptive histogram equalization (middle panel) followed by a background detection correction (right) that normalized intensities across the depth of the image. The bottom row shows the segmentation of the upper and lower regions of the grayscale image into solid material (red) and pores (gray). The 3D reconstructions for these two sub-regions were then merged using the ‘Attach’ function in GeoDict^®^ to create a single 3D reconstruction of the pore space, as shown in the lower right image. To verify that the reconstructed volumes preserved the expected global membrane structure, we evaluated the membrane porosity and the pore size distribution of the reconstructed geometry. The clean membrane exhibited a porosity that decreased slightly from approximately 0.40 near the inlet to about 0.35 toward the size-selective exit, with a corresponding reduction in the mean pore size, both consistent with the known asymmetric structure of the Viresolve^®^ Pro membrane. A more detailed analysis of the porosity and pore size distribution is provided in Section 3.4.

### 3.2. Validation of 3D Reconstructions

The detailed 3D pore space for the clean and fouled Viresolve^®^ Pro membranes were imported into GeoDict^®^ to evaluate the membrane permeability, the ratio of the volumetric flow rate to the transmembrane pressure difference divided by the membrane area, by solution of the incompressible Stokes equations. The calculated permeability of the 2.4 µm region that was imaged near the exit of the clean membrane is 5.9 L/m^2^/h/kPa; the permeability of the full membrane (140 µm in total thickness) would be expected to be somewhat less than this due to the resistance provided by rest of the pore structure. However, the bulk of the membrane resistance is provided by the small pores in the exit region of the membrane; the pore size in the Viresolve^®^ Pro increases by more than 5-fold over the first 20 µm near the filter exit [12]. For comparison, the experimentally measured permeability for the membranes examined in this study was 4.1 ± 0.2 L m^−2^ h^−1^ kPa^−1^, while Fallahianbijan et al. [12] reported a permeability of 5.8 L m^−2^ h^−1^ kPa^−1^ for a different lot of the Viresolve^®^ Pro membrane. These values are within the expected lot-to-lot variability reported for these membranes and demonstrates that the reconstructed volume properly captures the key structural features of the asymmetric Viresolve^®^ Pro membrane.

The permeabilities of the membranes fouled to different levels of flux decline (J/J_0_) are summarized in Table 1. For the membrane fouled to a 40% flux decline (J/J_o_ = 0.6) using a 1 g/L solution of IgG, the average permeability from two independently reconstructed regions of the membrane is 3.5 ± 0.6 L/m^2^/h/kPa, corresponding to J/J_0_ = 0.59 ± 0.08, which exactly matches the experimental value. A separate Viresolve^®^ Pro membrane was fouled to the same extent using a 0.1 g/L solution of IgG. The simulated permeability determined from two reconstructions of the pore space (from different locations within the same fouled membrane) is 2.9 ± 0.6 L/m^2^/h/kPa, corresponding to J/J_0_ = 0.49 ± 0.09. The calculated permeability for a single reconstruction of the membrane fouled to a 90% flux decline yields J/J_0_ = 0.33, which is 3x the experimental value. These differences between the simulated and measured permeabilities are due to a number of factors. First, it is important to note that the 3D reconstructions of the clean and fouled membranes were obtained with different membranes; the expected membrane-to-membrane variability in permeability is typically ±20%. Second, the 7 nm voxel resolution may be unable to fully characterize the geometry/flow in the narrowest pore constrictions, which would be particularly significant in the heavily fouled membranes due to protein deposition within the pore space. Third, we expect that the fouling pattern within the membrane will be heterogeneous, with the presence of some localized regions that are almost completely blocked while others remain relatively open. This heterogeneity may be more pronounced due to the low concentration of IgG (0.1 g/L) used in these experiments. Since the reconstructed field of view (≈2.4 µm) represents a small section of the membrane, regions adjacent to it may contain more extensive pore blockage that would further restrict the flow.

### 3.3. Particle Capture

In addition to evaluating the permeability, GeoDict^®^ was also used to simulate particle transport and capture within the clean and fouled membranes. Electrostatic and hydrophobic interactions were not included, because the goal was to evaluate how changes in pore structure and connectivity influence permeability and size-based capture behavior. 30 batches of spherical 20 nm particles were introduced into the membrane (25 particles per batch, giving a total of 750 particles). Particles were introduced randomly 100 nm above the inlet to the 3D reconstruction of the membrane. All 750 particles in the simulation were captured within the reconstruction; no particles were detected in the filtrate collected from the exit of either the clean or fouled Viresolve^®^ Pro membranes. The log reduction value (LRV) was calculated from the simulated particle transport as

(1) $$LRV={log}_{10}\left(\frac{N_{in}}{N_{out}}\right)$$

where $N_{in}$ and $N_{out}$ are the numbers of particles entering and exiting the reconstructed volume, respectively. In this case, with all 750 particles retained, we can conclude that the simulated *LRV* is ≥2.87 (with the lower limit evaluated assuming that the 751st particle was able to pass through the membrane).

To improve the statistical reliability of the calculated virus removal, additional simulations were performed in which 100 “ghost” particles were added to each batch. These ghost particles were tracked in the same way as real particles (until capture or transmission) but were then removed so that they do not affect the flow in subsequent batches. This made it possible to simulate the behavior of many more particles without unreasonable computational demands. All 3750 particles were retained by the membrane, corresponding to a simulated *LRV* ≥ 3.57. This high degree of virus retention is consistent with experimental measurements from our previous work showing *LRV* > 4.3 for both the clean and fouled Viresolve^®^ Pro membranes using ϕX174 bacteriophage with a diameter of approximately 25 nm as a model virus [1].

**Figure 4:**
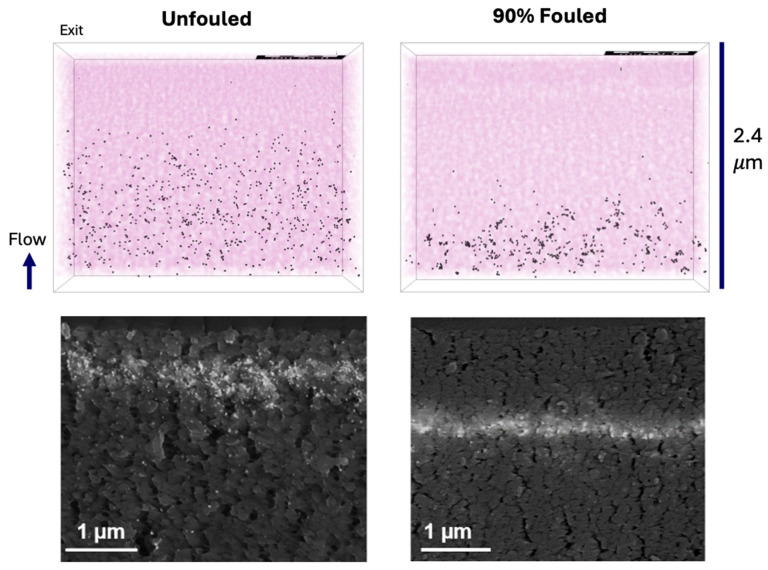
**Top panel**: Simulated capture profiles for 20 nm nanoparticles in unfouled and 90% fouled Viresolve^®^ Pro membranes after filtration of 30 batches of 25 particles. **Bottom panel**: Cross-sectional SEM images of 20 nm gold nanoparticle capture in unfouled and 90% fouled Viresolve^®^ Pro membranes (adapted from [12]). Fouling performed by filtration of IgG solutions.

The top panel of Figure 4 shows the simulated capture positions for the 20 nm particles in the unfouled (left panel) and 90% fouled (right panel) reconstructions of the Viresolve^®^ Pro membranes. Flow is from bottom to top in all simulations, with the bottom surface corresponding to the entrance of the reconstructed volume and the top surface corresponding to the size-selective exit layer of the membrane. The capture locations in the unfouled membrane were distributed across most of the depth of the 3D reconstruction. In contrast, the particles in the fouled membrane were captured closer to the entrance of the 3D reconstruction (further from the filter exit), with fewer particles able to penetrate deeply into the porous structure. This difference reflects changes in the effective pore size and local flow pathways caused by protein fouling. In the clean membrane, the fluid flow remains well connected through the full thickness of the reconstruction, allowing particles to move deeper before being captured. In the fouled membrane, blockage of the small exit-side pores restricts access to the deeper regions of the structure near the filter exit.

For comparison, the bottom panel of Figure 4 shows cross-sectional SEM images of the capture of 20 nm gold nanoparticles in the unfouled (left panel) and 90% fouled (right panel) Viresolve^®^ Pro membranes, as obtained in previous experimental studies by Fallahianbijan et al. [12]. The experimental results also show a shift in the mean capture location for the fouled membrane relative to that of the clean membrane, with the mean distance shifted further upstream (away from the filter exit) by approximately 1 µm. The experimental capture zones of gold nanoparticles (bottom panel) appear narrower than seen in the simulations (top panel), which may reflect the inherent limitations associated with the 7 nm voxel size in the 3D reconstructions or to the presence of electrostatic/hydrophobic interactions on the experimental results, which could result in particle capture even in regions where the pore size is slightly greater than the particle size.

**Figure 5:**
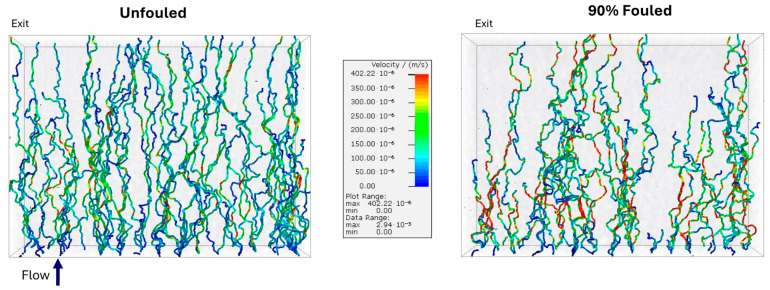
Simulated trajectories of non-interacting point particles through the unfouled (**left**) and 90% fouled (**right**) Viresolve^®^ Pro membranes, color-mapped by velocity.

Additional insights into the flow and transport behavior were obtained from the trajectory simulations in GeoDict^®^. Figure 5 shows the trajectories of a set of 300 massless point particles introduced within the unfouled (left) and 90% fouled (right) Viresolve^®^ Pro reconstructions. The particles were seeded in a uniform 20 × 15 grid above the entrance to the reconstructions using the Inflow placement setting in GeoDict^®^.

The trajectories in the unfouled membrane follow longer, more vertically directed paths that extend through the full depth of the membrane. The trajectories in the fouled membrane are noticeably shorter and more laterally deflected, with many terminating in the upper portion of the structure, indicating a reduction in the number of connected flow paths and an increase in the effective tortuosity of the remaining open channels. The shortest and most tortuous trajectories correspond to regions where protein deposition has blocked or constricted the small exit-side pores, which is consistent with the distribution of capture sites seen in Figure 4.

Although the overall permeability decreases upon fouling, there are regions within the fouled membrane that show even higher local velocities than obtained with the clean membrane due to the change in the underlying pore size distribution. These differences show that protein fouling reduces the number of connected flow channels, forcing the liquid to move through narrower, high-velocity, and more tortuous pathways near the entrance. This restricted flow pattern explains the lower permeability observed experimentally and the shift in particle capture toward upstream regions of the membrane.

### 3.4. Porosity and Pore Size Distribution

**Figure 6:**
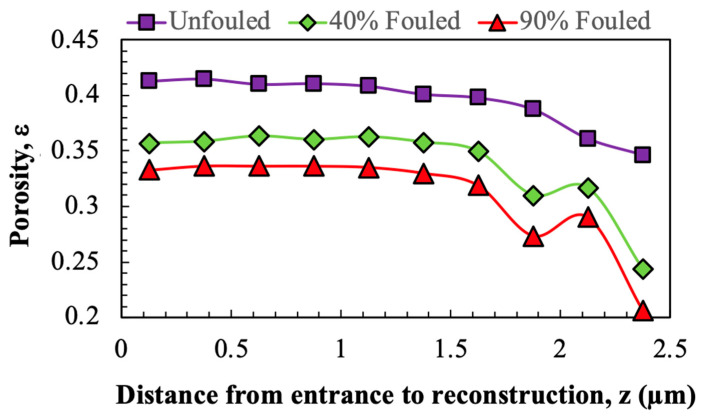
Porosity profiles through the unfouled, 40% fouled, and 90% fouled Viresolve^®^ Pro membranes determined directly from the 3D reconstructions. Filter exit is at z = 2.4 µm.

Figure 6 shows the porosity profiles for the unfouled, 40% fouled, and 90% fouled Viresolve^®^ Pro membranes determined directly from the 3D pore reconstructions. In each case, the porosity was calculated from the number of voxels located in the pore space divided by the total number of voxels in 250 nm (0.25 µm) horizontal slices through the reconstructions, with the values plotted at the depth of the mid-point of the slice (as measured from the inlet to the reconstruction). The porosity decreases towards the filter exit (at z = 2.4 µm), particularly over the last 1 µm of the membrane, consistent with the asymmetric structure of the Viresolve^®^ Pro membrane. The porosities of the Viresolve^®^ Pro after IgG fouling are uniformly smaller than those for the clean membrane, with a greater reduction in porosity after fouling to a 90% flux decline, as expected. The gradual drop in porosity across the depth of the reconstruction also indicates that fouling is not confined to the exit layer but extends into the upper portion of the porous structure. Although the entrance region appears similar in the 2D SEM images (Figure 1), the porosity values in Figure 6 were obtained from the full 3D voxel reconstructions, which capture depth-dependent changes that are not visible in single cross-sections of the highly asymmetric Viresolve^®^ Pro membrane. The entrance region contains larger pores with high void volume, with protein deposition in this region causing only a small relative change in porosity. In contrast, the pores near the filter exit are much smaller, with protein deposition in these narrower pores leading to a greater reduction in porosity.

**Figure 7:**
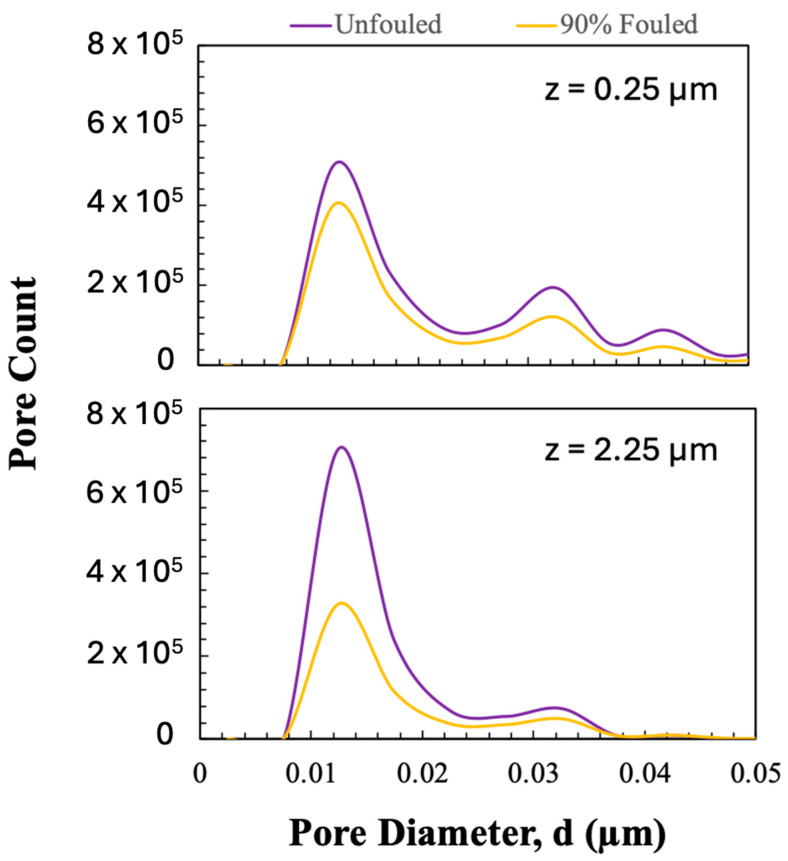
Pore size distribution in the unfouled and 90% fouled Viresolve^®^ Pro membranes near the entrance (z = 0.25 µm) and size-selective exit (z = 2.25 µm) of the 3D reconstruction.

The pore size distribution of the unfouled and fouled membranes were evaluated in MATLAB based on the Euclidean distance map, which calculates the pore size based on the size of the largest sphere that can be accommodated at every point within the pore space. The calculated pore size distributions for a slice obtained near the entrance (upper panel) and exit (lower panel) of the 3D reconstructions are shown in Figure 7. The pore size distributions at z = 0.25 µm appear tri-modal, with peaks around 13, 32, and 43 nm; the peak around 43 nm is absent in the distributions near the filter exit (bottom panel), consistent with the pore size gradient in the Viresolve^®^ Pro membrane. This trimodal distribution is due to the finite (7 nm) size of the voxels in the 3D reconstructions, which limits the ability to fully capture the narrowest constrictions in the membrane. Each voxel was taken as either void or solid based on its grayscale intensity; thus, changes in pore size tend to occur in multiples of the voxel size, leading to the discrete peaks seen in Figure 7. Even so, the observed changes in pore size and porosity align well with the measured permeability and particle capture behavior, indicating that the main effects of fouling are still well represented at this resolution. For the fouled membrane, the pore size distribution in the region near the entrance of the reconstruction shows a small but nearly uniform reduction in the pore count. In contrast, the pore size distribution near the filter exit in the fouled membrane shows a sharp reduction in the number of smaller pores, with minimal change in the number of larger pores, consistent with preferential blockage of the smaller pores in the distribution.

## 4. Conclusions

Our results show for the first time that FIB-SEM tomography can quantify the effects of protein fouling on the 3D pore space and in turn the performance of virus removal filtration membranes. Significant optimization of the FIB-SEM imaging, alignment, and segmentation were required to minimize degradation and desorption of the deposited protein and ensure accurate 3D reconstructions of the pore space. The calculated permeabilities of the clean and fouled membranes were in good agreement with experimental measurements, providing validation of the methodology.

The reconstructed volumes for the unfouled Viresolve^®^ Pro membrane show the expected asymmetric structure, with a thin region having very small pores located right near the exit of the membrane. The fouled membranes show a bright, dense band right near the exit surface, indicating significant protein deposition in this region of the membrane. However, the calculated porosity and pore size distributions also show small but significant changes in the region further upstream, suggesting that IgG fouling occurs throughout a much greater depth of the membrane.

The 3D reconstructions of the pore space were imported into GeoDict^®^, enabling calculation of the detailed velocity profiles and the location and extent of nanoparticle capture. The simulations indicate that the clean and fouled membranes both provide very high levels of virus retention (*LRV* ≥ 3.57), consistent with independent experimental measurements. Membrane fouling shifted the location of virus capture to a region further upstream of the size-selective skin layer, in good agreement with previous observations of gold nanoparticle capture in clean and fouled Viresolve^®^ Pro membranes. This shift was due to a significant reduction in the number of small pores near the filter exit, consistent with the preferential blockage of these small pores by the IgG (or IgG dimers or oligomers). This shift in particle capture could potentially alter the overall degree of virus removal and/or the response to process disruptions, which have been previously shown to impact virus retention [1]. These data provide the first direct measurements of the changes in the 3D pore structure due to protein fouling, providing a general strategy that can be used to obtain important fundamental insights into the effects of protein fouling on the performance of different membranes or membrane processes.

## Figures and Tables

**Table 1:** Simulated permeabilities and normalized flux ratios (J/J_0_) for Viresolve^®^ Pro membranes fouled to different levels of flux decline. Simulations for the 40% fouled membranes were averaged across two independently reconstructed regions; the 90% fouled condition was based on a single reconstruction.

Fouling Condition	Simulated Permeability (L/m^2^/h/kPa)	Average Simulated J/J_0_ Ratio	Experimental J/J_0_ Ratio
40% fouled (1 g/L)	3.5 ± 0.6	0.59 ± 0.08	0.6
40% fouled (0.1 g/L)	2.9 ± 0.6	0.49 ± 0.09	0.6
90% fouled (1 g/L)	2.0	0.33	0.1

## Data Availability

The data presented in this study are available on request from the corresponding author.

## Supplementary Materials

The following supporting information can be downloaded at: https://www.mdpi.com/article/10.3390/membranes15120369/s1, Figure S1: Effect of gravity and least-squares alignment on the 3D reconstructions. Figure S2: Effect of least-squares alignment on the 3D reconstruction of an inclined cylinder. (a) shows an inclined object in 3D, (b) shows how it appears across slices when uncorrected, and (c) shows how least-square alignment flattens the structure by forcing alignment across slices. Figure S3: SEM images obtained from repeat scans at a fixed location without milling, confirming that electron beam exposure causes desorption of deposited protein.
