# Supplementary material for: Quantitative Analysis of Protein Fouling in Virus Removal Filtration Membranes Through Electron Tomography
Source: Membranes (Basel). 2025 Dec 2;15(12):369. doi: 10.3390/membranes15120369 (PMC12734478; doi:10.3390/membranes15120369)
Supplement: Supplementary file 1 [file membranes-15-00369-s001.zip › membranes-3981703-supplementary.pdf]

membranes MDPI

Article

Quantitative Analysis of Protein Fouling in Virus Filtration Membranes through Electron Tomography

Mohammad A. Afzal[1], Kaitlyn P. Brickey[1], Enrique D. Gomez,[1,2,3] and Andrew L. Zydney[1*]

1 Department of Chemical Engineering, The Pennsylvania State University, University Park, PA 16802
2 Department of Materials Science and Engineering, The Pennsylvania State University, University Park, PA 16802
3 Materials Research Institute, The Pennsylvania State University, University Park, PA 16802
* Author to whom correspondence should be addressed: alz3@psu.edu

Supplementary Information

Several different approaches were initially explored to align and segment the FIB-SEM images. Figure S1 shows results obtained with gravity and least-squares alignment. Gravity alignment was unable to correct the vertical drift seen in the original reconstruction, while least-squares alignment led to vertical compression and cropping of key regions, including the carbon layer and fouling zone. These effects were particularly problematic in fouled Viresolve® Pro samples, where distortion / cropping near the top surface of the membrane made it impossible to quantify the effects of protein fouling on the 3D pore space.

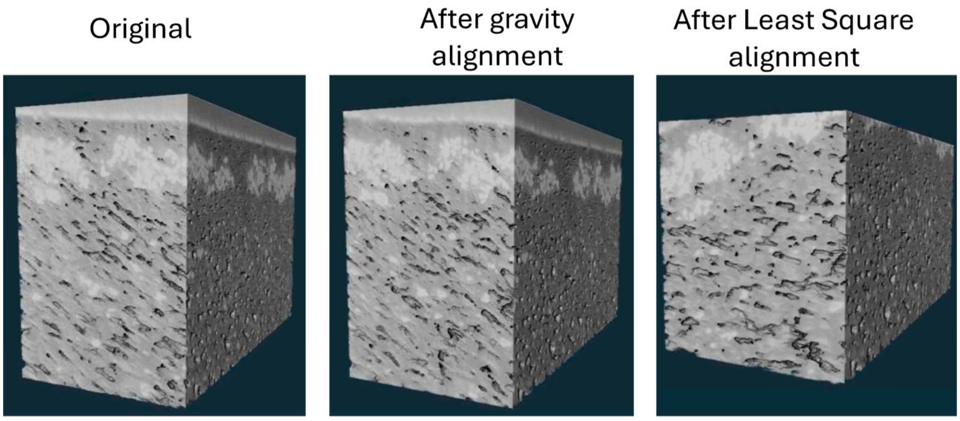

Figure S1. Effect of gravity and least-squares alignment on the 3D reconstructions.

The problem with the least-squares alignment method lies in its dependence on the lateral similarity of features between consecutive slices. This assumption breaks down in cases where structural features are tilted or asymmetric. This is discussed in some detail by Yuan et al. [21] and is illustrated in Figure S2. In panel (a), a cylindrical object is positioned at an angle, mimicking the geometry of an inclined pore. In panel (b), the object appears correctly as a series of laterally shifted ellipses across consecutive slices. However, in panel (c), least-squares alignment forces these ellipses into the same lateral position, flattening the object and truncating its edges. A similar distortion was observed in the membrane datasets when standard least-squares alignment was applied.

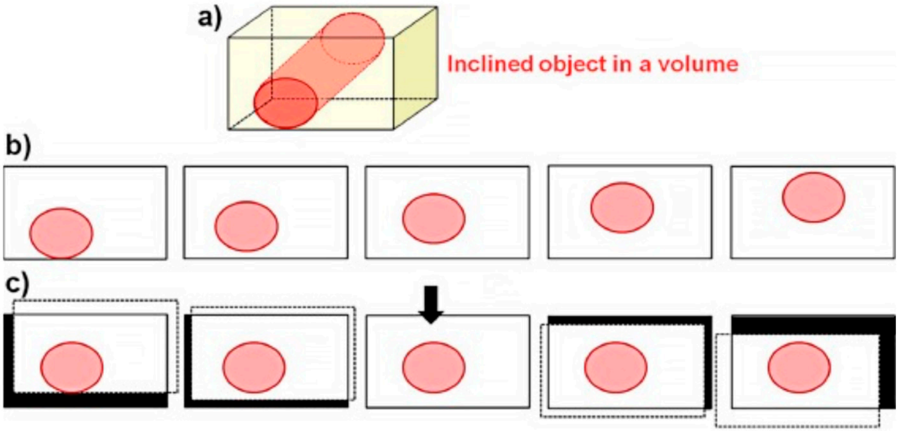

Figure S2. Effect of least-squares alignment on the 3D reconstruction of an inclined cylinder. (a) shows an inclined object in 3D, (b) shows how it appears across slices when uncorrected, and (c) shows how least-square alignment flattens the structure by forcing alignment across slices. Adapted from Yuan et al. (2021).

As discussed in the text, initial SEM images showed changes in intensity that appeared to be associated with the desorption / degradation of the deposited IgG. This was explored by performing repeat SEM scans at a fixed location without ion beam milling. The initial image (far left) shows significant protein deposition right beneath the upper surface of the membrane, i.e., immediately upstream of the filter exit. The subsequent scans show significantly less protein and a large increase in porosity due to progressive removal of protein upon repeated exposure to the electron beam under vacuum conditions. This effect was largely eliminated by using a lower current to protect these beam-sensitive samples.

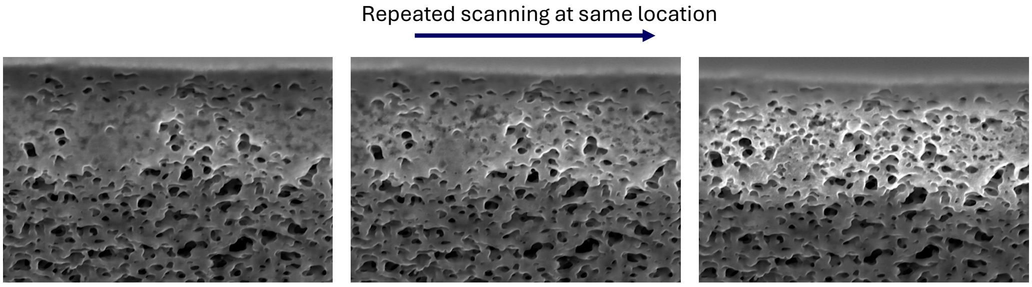

Figure S3. SEM images obtained from repeat scans at a fixed location without milling, confirming that electron beam exposure causes desorption of deposited protein.

Disclaimer/Publisher's Note: The statements, opinions and data contained in all publications are solely those of the individual author(s) and contributor(s) and not of MDPI and/or the editor(s). MDPI and/or the editor(s) disclaim responsibility for any injury to people or property resulting from any ideas, methods, instructions or products referred to in the content.
